# The Classification of Pellagra

**Published:** 1911-09

**Authors:** Hansell Crenshaw

**Affiliations:** Atlanta, Ga.


### THE CLASSIFICATION OF PELLAGRA.

By Hansell Crenshaw, M. D., Atlanta, Ga.

The tendency of specialists to appropriate unto themselves diseases of doubtful classification is unquestionable. It is only natural for the neurologist, for example, to see in any obscure condition the nervous features more clearly than the other features of equal or greater prominence. We are prone to see through a magnifying medium the things in which we are interested and for which we are continually on the watch.

Thus, as the result of a thoroughly sincere though warped perspective, the special workers in skin diseases have appropriated pellagra as essentially a dermal affection. At the same time the gastro-enterologists have concluded that it is a disturbance of nutrition, and now comes the neurologist with the claim that both ante-mortem and post-mortem findings place this great plague incontrovertably among the acknowledged diseases of the nervous system.

Let us examine briefly the ground upon which each specialist bases his claim to pellagra. As far as the writer is able to learn, the only reason given for calling pellagra a skin disease is the fact that in many cases—not all—a characteristic eruption appears on the backs of the wrists and now and then at other points on the body. But do not characteristic skin lesions appear as incidents in the course of such decidedly nervous maladies as cerebro-spinal meningitis, herpes zoster and other trophic neuroses?

The case for the gastro-enterologist is somewhat stronger, [illegible] as prolonged diarrhoea and gastric disturbances make up more or less fundamental features of the disease. Yet, these symptoms, as in the case of the skin lesions, may be attributable to trophic and vaso-motor disturbances originating in, or at least through, the nervous system.

But turn, now, to the omnipresence of nervous and mental symptoms in pellagra. The prodromal stage of this disease usually lasting several weeks is characterized chiefly by depression, vertigo and headache—all expressions of an abnormal nervous system. Consider next the prominence and persistence of the melancholic phase of the disease and of the frequent lapse of pellagrins into melancholia proper. Observe furthermore the ataxia especially of the upper extremities and the spasticity and muscular weakness of the lower extremities which form so constant a part of the clinical picture of pellagra.

From four to ten per cent. of all pellagrins, we are told, are met with in asylums for the insane.

Most significant of all, however, are the post-mortem reevlations. Autopsies upon classic cases of pellagra invariably reveal evidence of meningitis, both leptomeningitis and pachymeningitis, sclerosis of the brain and cord, and atrophy of the anterior cornual cells.

"The cord lesions," say Church and Peterson, "consist of a leptomeningitis, often with much thickness, and even with the formation of osseous plaques. In the cord itself, the anterior cornual cells are frequently atrophied and pigmented. There is commonly a posterolateral sclerosis. This affects the columns of Goll and Burdach, mainly in the upper cord levels, but spares the root-zone of the postero-external column. The crossed pyramidal tract, especially the lower portion, is also sharply sclerosed, the direct cerebellar tract usually escaping.

"The spinal symptoms correspond. Ataxia is most marked in the upper extremities; spasticity is pronounced in the lower limbs. The iridian reflexes are spared, and cutaneous sensibility is not much affected. Strangely, in spite of the usual changes in the anterior cornual cells, muscular atrophy is insignificant. The disease, clinically and anatomically, presents much resemblance to paretic dementia."

Of the medley of speculation now playing upon the cause of pellagra, this much may be stated as established: Pellagra is an intoxication, and the toxic sucstance, whatever it is, brings about a degeneration of the tissues, but particularly a degeneration of the nervous tissues. Both grey matter and white matter seem to be more susceptible to the destroying influence of the pellagrous toxin than are any other tissues of the body.

Until the mouldy maize theory of the disease is convincingly disproved (if it ever is), pellagrins should be taken off of corn-meal and corn-containing foods of any sort; and arsenic, iron and calcium sulphide in varying combinations and alternations should be pushed. Whether the good which results from the use of these drugs is due to any more specific effect than their general tonic properties is more than doubtful; and, considering the schlerotic and other changes in the tissues, the iodides ought, *a priori*, to come nearer striking the root of the damage than either arsenic, iron, or the sulphide.

As in the case of tabes, the most we can hope to do in well-advanced cases of pellagra is to arrest the disease. No power, skill, or ingenunty of man will ever restore the integrity of a single neuron that has been destroyed. And when the smoke of battle between zeist and antizeist shall have cleared up; when the sand-fly theory and Buffalo gnat hypothesis shall have taken wing and flown away; and when oleagenous speculation shall have evaporated from the cold face of fact—then shall we in all probability find ourselves confronted by a trophic psycho-neurosis, preventable if intactible, amenable to arrest if not among the actually curable ills of man.

1027 Candler Bldg.

